# Metagenomic pipeline for identifying co-infections among distinct SARS-CoV-2 variants of concern: study cases from Alpha to Omicron

**DOI:** 10.1038/s41598-022-13113-4

**Published:** 2022-06-07

**Authors:** Jose Arturo Molina-Mora, Estela Cordero-Laurent, Melany Calderón-Osorno, Edgar Chacón-Ramírez, Francisco Duarte-Martínez

**Affiliations:** 1 Centro de Investigación en Enfermedades Tropicales (CIET) and Facultad de Microbiología, Universidad de Costa Rica, San José, Costa Rica; 2 Instituto Costarricense de Investigación y Enseñanza en Nutrición y Salud (INCIENSA), Tres Ríos, Cartago Costa Rica

**Keywords:** Genome informatics, Software, Cell biology, Computational biology and bioinformatics, Genetics, Microbiology, Molecular biology, Molecular medicine, Pathogenesis, Infectious diseases

## Abstract

Concomitant infection or co-infection with distinct SARS-CoV-2 genotypes has been reported as part of the epidemiological surveillance of the COVID-19 pandemic. In the context of the spread of more transmissible variants during 2021, co-infections are not only important due to the possible changes in the clinical outcome, but also the chance to generate new genotypes by recombination. However, a few approaches have developed bioinformatic pipelines to identify co-infections. Here we present a metagenomic pipeline based on the inference of multiple fragments similar to amplicon sequence variant (ASV-like) from sequencing data and a custom SARS-CoV-2 database to identify the concomitant presence of divergent SARS-CoV-2 genomes, i.e., variants of concern (VOCs). This approach was compared to another strategy based on whole-genome (metagenome) assembly. Using single or pairs of sequencing data of COVID-19 cases with distinct SARS-CoV-2 VOCs, each approach was used to predict the VOC classes (Alpha, Beta, Gamma, Delta, Omicron or non-VOC and their combinations). The performance of each pipeline was assessed using the ground-truth or expected VOC classes. Subsequently, the ASV-like pipeline was used to analyze 1021 cases of COVID-19 from Costa Rica to investigate the possible occurrence of co-infections. After the implementation of the two approaches, an accuracy of 96.2% was revealed for the ASV-like inference approach, which contrasts with the misclassification found (accuracy 46.2%) for the whole-genome assembly strategy. The custom SARS-CoV-2 database used for the ASV-like analysis can be updated according to the appearance of new VOCs to track co-infections with eventual new genotypes. In addition, the application of the ASV-like approach to all the 1021 sequenced samples from Costa Rica in the period October 12th–December 21th 2021 found that none corresponded to co-infections with VOCs. In conclusion, we developed a metagenomic pipeline based on ASV-like inference for the identification of co-infection with distinct SARS-CoV-2 VOCs, in which an outstanding accuracy was achieved. Due to the epidemiological, clinical, and molecular relevance of the concomitant infection with distinct genotypes, this work represents another piece in the process of the surveillance of the COVID-19 pandemic in Costa Rica and worldwide.

## Introduction

The COVID-19 pandemic, caused by the SARS-CoV-2 virus, has affected 282 million people worldwide and 570,000 people in Costa Rica by December 2021. The genomic sequencing approach is one of the hallmarks in the management of COVID-19 to follow up virus evolution and spread across the globe almost in real-time, unlike other pandemics^1^. Thus, since the emergence of the virus, efforts have been made to map the genetic diversity of the virus and to identify genotypes with a possible selective advantage^2^.

The SARS-CoV-2 genotypes, which share several common mutations and are expected to have similar biological properties, can be classified as clades, PANGOLIN lineages, or variants depending on the nomenclature system. These versions of the SARS-CoV-2 virus have been reported each time faster during the last year in part due to the increased mutation rate of the virus over time^3^. Out of the thousands of lineages that have been reported at the end of the year 2021, the World Health Organization (WHO) has recognized five of those divergent genotypes as a variant of concern (VOC), mainly due to the increased transmissibility and/or a capacity to evade inhibition by neutralizing antibodies. The divergent VOCs, namely Alpha (lineage B.1.1.7), Beta (B.1.351), Gamma (P.1), Delta (B.1.617.2), and Omicron (B.1.1.529) variants, have been initially reported in specific geographic regions, but rapidly were spread to multiple locations worldwide^3–5^. Other genotypes, such as the variants of interest (VOI) or variants under monitoring (VUM) have been also reported, which are still under study owed to possible changes in the patterns of transmission, severity, clinical manifestations, mortality, or vaccine effectiveness^6,7^.

Because of the amount and features of circulating variants, epidemiological surveillance of the pandemic must include the analysis of concomitant infections (co-infection) with different SARS-CoV-2 genomes^8^. Co-infection can be described as the occurrence of a re-infection when a first infection was not yet cured^9^ or the horizontal transmission of multiple genotypes^11^. Estimation of frequency and the study of effects of co-infections are relevant not only for the management of the disease at the personal (symptoms) or population (transmission) level but also for the molecular surveillance of possible risky events of recombination that can be triggered^10^. However, the incidence of concomitant mixed infections with different genotypes has not been extensively reported^11,12^. Some studies have reported up to 2.6–8% of COVID-19 cases as co-infections^2,3,12^, but a more specific and confident analysis found that 0.18% of cases were concomitant infections^13^.

Regarding the bioinformatic strategies, only a few studies have implemented analyses to detect co-infections by SARS-CoV-2 genomes^2,10–12^. These pipelines are based on the identification of haplotypes (sequences of each genome in the concomitant infection) using haplotype reconstruction programs^2,10–12^. However, haplotype identification is usually used to detect co-infections with related but distinct viruses and performs poorly for close genomes^29^. The only specific pipeline to identify co-infections by divergent SARS-CoV-2 viruses was recently developed by^13^.

In this context and as part of the epidemiological surveillance of the pandemic in Costa Rica, we now present a new pipeline based on metagenomic analyses to detect co-infections with divergent SARS-CoV-2 viruses, specifically with VOCs. After sequencing and pre-processing, the workflow follows the inference of multiple fragments similar to amplicon sequence variant (ASV-like) from sequencing data and a taxonomy assignment with a custom database of SARS-CoV-2 sequences. Thus, this study aimed to develop a pipeline to identify co-infections with divergent SARS-CoV-2 genomes using an ASV inference approach.

## Methods

### General strategy

**Figure 1:**
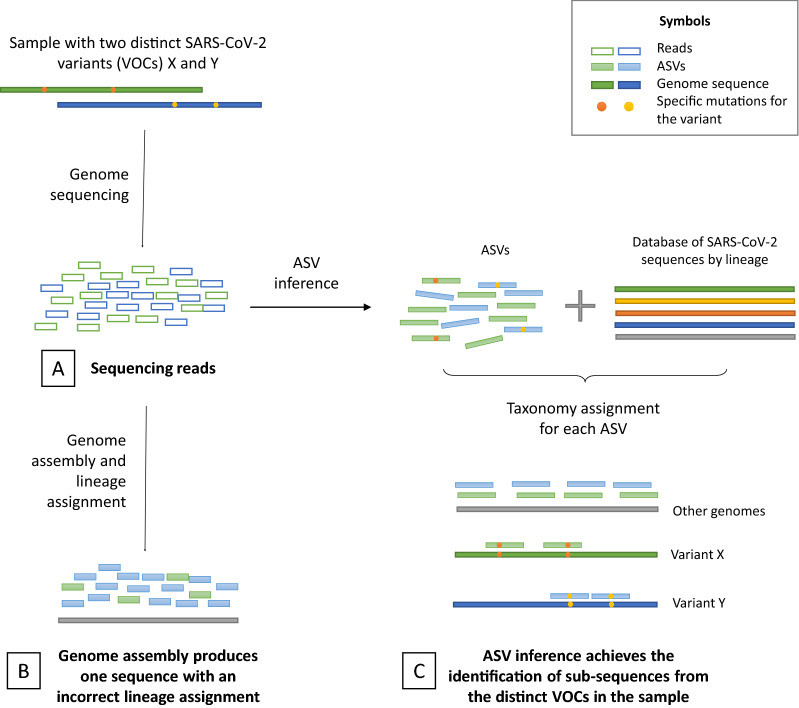
Conceptual design of the approach to identify co-infection by SARS-CoV-2 VOCs. Samples with two distinct VOCs (X and Y) of the SARS-CoV-2 virus are sequenced (**A**). Using a strategy for the whole-genome assembly, a single and misclassified sequence is obtained (**B**). In contrast, the correct identification of the two VOCs is achieved when an ASV inference is implemented, with the use of a custom database of SARS-CoV-2 for the taxonomy assignment. In this process, specific ASV are recognized for the VOCs, while shared ASVs among all the genomes are assigned to other sequences.

To identify cases of COVID-19 with a co-infection with two distinct SARS-CoV-2 VOCs, we implemented a strategy using genome sequencing data and two different pipelines (Fig. 1). Sequencing data of samples with different SARS-CoV-2 lineages (one or two lineages, including VOCs) were obtained (Fig. 1A). A first strategy was the whole-genome assembly, in which a metagenomic assembler was used to build the genome sequence(s) in the sample. After the lineage assignment, genome sequences in each sample were classified into VOC classes (Alpha, Beta, Gamma, Delta, Omicron, or non-VOC) and this prediction was compared to the known or expected categories (Fig. 1B).

In a second approach, sequencing data were used for the analysis with sequences at single-nucleotide resolution, the ASV-like calling strategy. In this case, lineage assignment was not directly done but ASVs were mapped to a custom database of SARS-CoV-2 genomes sequences. For genome sub-sequences that are shared among all the lineages, the corresponding ASV are expected to map multiple sequences, including the non-VOC genomes if they are provided first. However, ASVs carrying specific mutations of the VOC are expected to only map to the genome of the variant. Thus, for each genome sequence in the database, the mapping ASVs were counted to assign the sample to a VOC category (Fig. 1C). The prediction of these classes was compared to the expected results to assess the performance of the pipeline.

### Clinical isolates and genome sequencing

Using the sample collection of Costa Rican cases of COVID-19 in the period between March 2020 and August 2021 as part of the genomic surveillance of the SARS-CoV-2 virus, 12 samples from distinct lineages were selected (single lineages in Table 2). Genotypes included VOCs and VOIs, as well as the regional lineage B.1.1.389 circulating in Costa Rica (Table 2).

Patients had been diagnosed in INCIENSA (Instituto Costarricense de Investigación y Enseñanza en Nutrición y Salud) or different public and private clinical laboratories by real-time reverse transcription-polymerase chain reaction (RT-PCR) using nasopharyngeal swab samples. The diagnosis was done using the guidelines of the Pan American Health Organization and the World Health Organization^14^, and the Ministry of Health of Costa Rica. All subsequent experiments and analyses were performed following Costa Rican guidelines and regulations.

Selected samples had a CT < 25 (cycle threshold in the PCR) and genome sequencing had been done in the local sequencing service of INCIENSA. Amplicons were obtained using the protocol by^15^. Sequencing libraries were prepared using the Illumina DNA Prep Kit (Illumina, San Diego, CA, USA) according to the laboratory standard operating procedure for pulsenet Nextera DNA flex library preparation (https://www.cdc.gov/pulsenet/pathogens/wgs.html). Paired-end sequencing was performed for each library on a MiSeq instrument using 500 cycles v2 chemistry cartridges (Illumina, San Diego, CA, USA).

### Pre-processing and ground-truth genotype

FastQC v0.11.7^16^ was used for the quality control of sequencing data. Trimmomatic v0.38^17^ was used for adapters removal and trimming of low-quality bases (Q < 30). Filtered reads were used to infer the ground-truth genotype, the de novo whole-genome assembly, and the ASV calling.

To identify the ground-truth (expected) genotype of the sequences, a reference-based genome assembly was implemented. BWA-MEM 0.7.5a-r405^18^ with default parameters was used to map reads to the reference genome NC_045512.2. Freebayes v1.3.1^19^ (parameters -p 1 -q 20 -m 60 -min-coverage 10 –V) was implemented to call variants. Low-confidence variants were removed using VCF_filter v3.2 (https://github.com/moskalenko/vcf_filter). Annotation of variants was done using SNPeff^20^. The genotype for each genome was allocated using the PANGOLIN lineages assigner version 3.1.17 (https://pangolin.cog-uk.io/). Based on the lineage, genome sequences were classified into the VOC classes (Alpha, Beta, Gamma, Delta, Omicron, or non-VOC) and the results were used as the ground-truth genotype (expected lineage or VOC class, Table 2).

Based on the SARS-CoV-2 genotypes found in the 12 samples (samples with a single lineage, Table 2), 14 new datasets were generated by combining sequencing data of two distinct cases (double lineages, Table 2). The ground-truth genotypes were inferred based on the individual genomes (expected genotypes, Table 2).

### Whole-genome (metagenome) assembly

To assemble the genome of cases with a single (12 samples) or double (14 samples) genotype, a de novo metagenomic assembler was implemented using the filtered sequencing reads. Megahit v1.1.3^21^ was used due to its ability to build sequences of an individual or multiple genomes^21,22^. Genome assembly was evaluated based on contiguity, completeness, and correctness using the 3C criterion^22,23^. The genotype for each genome was allocated using the PANGOLIN lineages assigner (https://pangolin.cog-uk.io/). Based on the lineage, the 26 genome sequences were classified into the VOC classes and the results were used as the prediction of this pipeline. The predicted genotypes were compared to the expected (ground-truth) genotypes (Table 2).

### ASV-like inference

To call ASVs for each sample, the DADA2 package^24^ was run using the R software. The standard protocol of this software for Illumina sequencing data was implemented (https://benjjneb.github.io/dada2/tutorial.html), in which the only modified step was the taxonomy assignment using a custom database (see below). Briefly, sequencing data (fastq files) were filtered by quality as error rates were calculated and removed from the dereplicated reads. Possible chimeric reads were identified and removed. Finally, taxonomy was assigned using the RDP Naive Bayesian Classifier algorithm against a custom SARS-CoV-2 database, and an error-corrected table of the abundances of ASVs was obtained. In addition, because of the nature of the ASV inference in which a consensus sequence is built and results are dependent on the iteration, we completed 5 repetitions of the analysis to verify the reproducibility of the results.

#### Custom SARS-CoV-2 database

To assign the taxonomy to the ASVs, nine SARS-CoV-2 genome sequences were incorporated into a custom database. Sequences corresponded to VOCs (which were retrieved from https://viralzone.expasy.org/9556) and other non-VOC genomes circulating in Costa Rica since March 2020, which are detailed in Table 1. Because of the use of the DADA2 pipeline, the database required a format like 16S-rRNA databases for amplicon-based metagenomics (16S-rRNA). Thus, the sequence name in the fasta file required a format including the organism and subtype, i.e., “ > SARS-CoV-2; VOC_class-lineage-ID” according to the data in Table 1. The SARS-CoV-2 genome database is provided as a supplementary file.

**Table 1:** SARS-CoV-2 sequences used to build the custom database to classify genome sequences into VOC categories based on ASV calling analysis.

ID	Lineage	VOC classes	Database
CRC-0381	B.1.1.519	Non-VOC	GISAID
CRC-0449	B.1.1.389	Non-VOC	GISAID
CRC-0493	B.1.525	Non-VOC	GISAID
CRC-0653	C36.3	Non-VOC	GISAID
MZ344997.1	B.1.1.7	Alpha	NCBI
MW598419.1	B.1.351	Beta	NCBI
MZ169911.1	P.1	Gamma	NCBI
MZ359841.1	B.1.617.2	Delta	NCBI
PI_ISL_6913995	B.1.1.529	Omicron	GISAID

### Assessment of the pipelines performance

To assess the performance of the two pipelines to identify co-infections, the genome assembly and the ASV calling, the predictions regarding the VOC class of each approach were compared to the expected genotype. A ROC (Receiver Operating Characteristics) analysis was implemented in R software (https://www.r-project.org/) using the ROCR package^25^. AUC (Area Under Curve) of the ROC curve, as well as the general accuracy of the predictions, were calculated for each pipeline.

### Estimation of occurrence of co-infections in Costa Rica

Using the ASV-like approach, we run the pipeline to identify possible co-infection with VOCs in COVID-19 cases in Costa Rica. The analysis was done using all the samples which had been locally sequenced (1021 cases) in the period October 12th–December 21th 2021. Samples were processed as described before.

### Ethical approval and consent to participate

This study was approved by INCIENSA (INCIENSA-DG-of-2020-174) and the scientific committee of CIET-UCR (No. 242-2020). Samples were collected for epidemiological surveillance according to the Costa Rican regulation Law Nº 8270 (May 17th, 2002), in which no additional consent was required for retrospective studies of archived and anonymized samples. All experiments were performed following Costa Rican guidelines and regulations.

## Results

**Figure 2:**
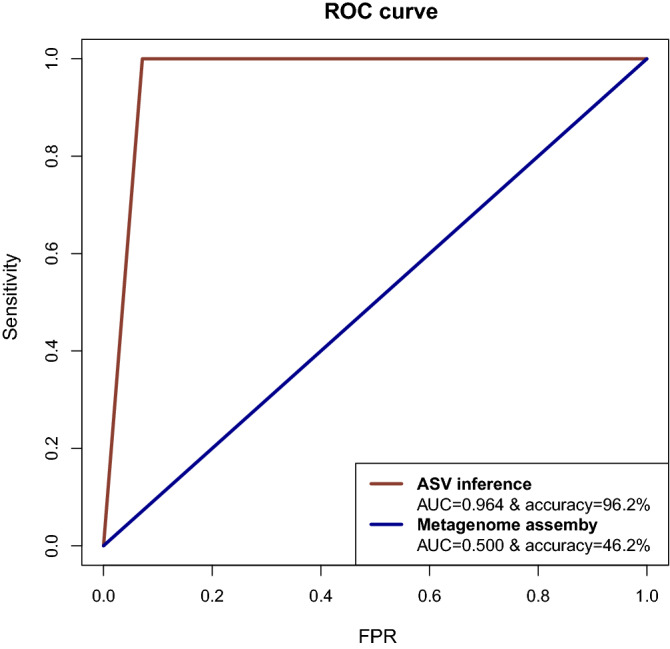
Performance of two distinct pipelines to identify co-infection by SARS-CoV-2 VOCs. Metrics for the whole-genome assembly, with AUC = 0.5 and accuracy = 46.2%, suggest no discrimination of the VOCs among samples, with a misclassification of all the cases with two variants. In contrast, the ASV-like inference was able to correctly identify VOCs in cases with one or two variants, with an outstanding performance according to the AUC = 0.964 and accuracy = 96.2%. FPR: False Positive Rate.

In order to identify cases of COVID-19 with a co-infection with two distinct SARS-CoV-2 VOCs, we implemented a strategy using genome sequencing data and two different pipelines (Fig. 1). First, a whole-genome analysis approach, which included the metagenome assembly, lineage assignment, and the classification into the VOC categories, resulted not suitable to identify co-infections with VOCs. With an accuracy of 46.2%, this strategy misclassified all the genome sequences for cases with two lineages, including VOCs, while only samples with a single lineage were properly identified (Table 2 and Fig. 2). The ROC analysis found a value of AUC = 0.500, revealing that the performance of the VOC assignment is equivalent to a classification by chance.

**Table 2:** Genome classification using a whole-genome assembly strategy with sequencing data for one or two variants of the SARS-CoV-2 virus (PERF: performance of the prediction regarding the expected class).

Samples		Expected genotype		Predicted genotype		PERF
Type	ID	Expected lineage	Expected VOC class	Predicted lineage	Predicted VOC class	
Single lineage	S1	B.1.1.389	Non-VOC	B.1.1.389	Non-VOC	✔
	S2	C.36.3	Non-VOC	C.36.3	Non-VOC	✔
	S3	P.2	Non-VOC	P.2	Non-VOC	✔
	S4	B.1.625	Non-VOC	B.1.625	Non-VOC	✔
	S5	B.1.429	Non-VOC	B.1.429	Non-VOC	✔
	S6	B.1.525	Non-VOC	B.1.525	Non-VOC	✔
	S7	B.1.1.519	Non-VOC	B.1.1.519	Non-VOC	✔
	S8	B.1.1.7	Alpha	B.1.1.7	Alpha	✔
	S9	B.1.351	Beta	B.1.351	Beta	✔
	S10	P.1	Gamma	P.1	Gamma	✔
	S11	AY.113	Delta	AY.113	Delta	✔
	S12	B.1.1.529	Omicron	B.1.1.529	Omicron	✔
Double lineage	D1 (S4 + S6)	B.1.625 + B.1.525	Non-VOC (+ Non-VOC)	B.1	Non-VOC	✘
	D2 (S1 + S8)	B.1.1.389 + B.1.1.7	Alpha (+ Non-VOC)	B.1.1	Non-VOC	✘
	D3 (S8 + S9)	B.1.1.7 + B.1.351	Alpha + Beta	B.1	Non-VOC	✘
	D4 (S8 + S10)	B.1.1.7 + P.1	Alpha + Gamma	B.1	Non-VOC	✘
	D5 (S8 + S11)	AY.113 + B.1.1.7	Alpha + Delta	B.1	Non-VOC	✘
	D6 (S8 + S12)	B.1.1.7 + B.1.1.529	Alpha + Omicron	B.1	Non-VOC	✘
	D7 (S9 + S11)	AY.113 + B.1.351	Beta + Delta	B.1	Non-VOC	✘
	D8 (S9 + S10)	P.1 + B.1.351	Beta + Gamma	B.1	Non-VOC	✘
	D9 (S9 + S12)	B.1.351 + B.1.1.529	Beta + Omicron	B.1	Non-VOC	✘
	D10 (S11 + S10)	AY.113 + P.1	Delta + Gamma	B.1	Non-VOC	✘
	D11 (S10 + S12)	P.1 + B.1.1.529	Gamma + Omicron	B.1	Non-VOC	✘
	D12 (S11 + S2)	AY.113 + C.36.3	Delta (+ Non-VOC)	B.1.629	Non-VOC	✘
	D13 (S7 + S11)	AY.113 + B.1.1.519	Delta (+ Non-VOC)	B.1	Non-VOC	✘
	D14 (S11 + S12)	AY.113 + B.1.1.529	Delta + Omicron	B.1	Non-VOC	✘

**Table 3:** Genome classification using an ASV inference strategy with sequencing data for one or two variants of the SARS-CoV-2 virus (PERF: performance of the VOC prediction regarding the expected VOC class).

Samples		Expected genotype		Predicted genotype		PERF
Type	ID	Expected lineage	Expected VOC class	Score by iterations	Predicted VOC class	
Single lineage	S1	B.1.1.389	Non-VOC	5	Non-VOC	✔
	S2	C.36.3	Non-VOC	5	Non-VOC	✔
	S3	P.2	Non-VOC	5	Non-VOC	✔
	S4	B.1.625	Non-VOC	5	Non-VOC	✔
	S5	B.1.429	Non-VOC	5	Non-VOC	✔
	S6	B.1.525	Non-VOC	5	Non-VOC	✔
	S7	B.1.1.519	Non-VOC	5	Non-VOC	✔
	S8	B.1.1.7	Alpha	5	Alpha	✔
	S9	B.1.351	Beta	5	Beta	✔
	S10	P.1	Gamma	5	Gamma	✔
	S11	AY.113	Delta	5	Delta	✔
	S12	B.1.1.529	Omicron	5	Omicron	✔
Double lineage	D1 (S4 + S6)	B.1.625 + B.1.525	Non-VOC (+ Non-VOC)	5	Non-VOC (+ Non-VOC)	✔
	D2 (S1 + S8)	B.1.1.389 + B.1.1.7	Alpha (+ Non-VOC)	4	Alpha (+ Non-VOC)	✔
	D3 (S8 + S9)	B.1.1.7 + B.1.351	Alpha + Beta	3	Alpha + Beta	✔
	D4 (S8 + S10)	B.1.1.7 + P.1	Alpha + Gamma	4	Alpha + Gamma	✔
	D5 (S8 + S11)	AY.113 + B.1.1.7	Alpha + Delta	4	Alpha + Delta	✔
	D6 (S8 + S12)	B.1.1.7 + B.1.1.529	Alpha + Omicron	5	Alpha + Omicron	✔
	D7 (S9 + S11)	AY.113 + B.1.351	Beta + Delta	5	Beta + Delta	✔
	D8 (S9 + S10)	P.1 + B.1.351	Beta + Gamma	5	Beta + Gamma	✔
	D9 (S9 + S12)	B.1.351 + B.1.1.529	Beta + Omicron	5	Beta + Omicron	✔
	D10 (S11 + S10)	AY.113 + P.1	Delta + Gamma	5	Delta + Gamma	✔
	D11 (S10 + S12)	P.1 + B.1.1.529	Gamma + Omicron	5	Gamma + Omicron	✔
	D12 (S11 + S2)	AY.113 + C.36.3	Delta (+ Non-VOC)	5	Delta (+ Non-VOC)	✔
	D13 (S7 + S11)	AY.113 + B.1.1.519	Delta (+ Non-VOC)	0	Beta	✘
	D14 (S11 + S12)	AY.113 + B.1.1.529	Delta + Omicron	5	Delta + Omicron	✔

**Figure 3:**
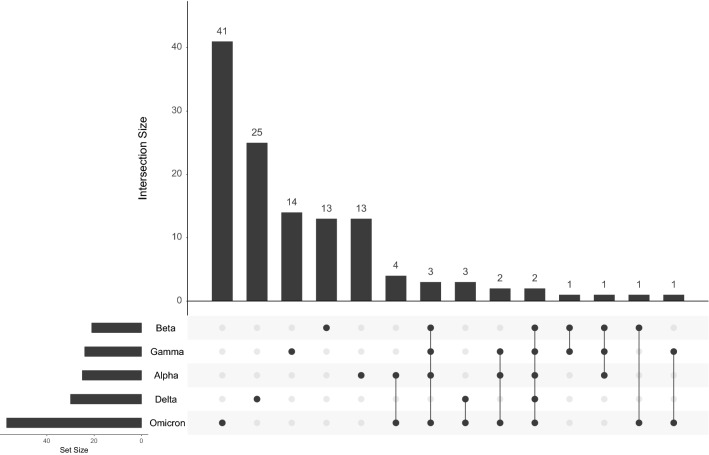
Comparison of the mutation profile of the SARS-CoV-2 VOCs. A few mutations are shared among the genotypes while most of them are exclusive to each genome, making it possible to map and track genome subsequences to identify the concomitant presence of VOCs.

To deal with this, a second metagenomic approach was implemented using an ASV-like calling strategy. A custom database of SARS-CoV-2 genomes sequences was created to assign the taxonomy of the ASV sequences into VOCs or Non-VOC genomes. During the standardization, it was determined that the identification of a VOC was possible if at least three ASV were mapped to the VOC genome. Thus, the presence of a specific VOC was determined if the number of total mapping ASVs was ≥ 3. This is in line with the profile of mutations for each VOC, in which a few mutations are shared and most of them are exclusive (Fig. 3).

According to Table 3, the ASV-inference pipeline was able to correctly classify all samples with two lineages but one (sample D13) into the VOC classes. In the same way, all the samples with a single genotype were correctly classified. As presented in the Supplementary file, the classification of the VOC class is consistent among the iterations for all the samples when the pipeline was run 5 times to assess reproducibility. In the case of the misclassified sample D13, the combination was done using the Delta variant and a case of the B.1.1.519 lineage. Results of the 5 iterations predicted a Beta variant in the sample.

When the profile of mutations was compared, 3 mutations (ORF1b-P314L, ORF8-S84L, and S-D614G) are shared by Beta, Delta, and B.1.1.519 genotypes, which made the ASV-like inference to incorrectly assign the subsequences to the Beta variant. However, this phenomenon was not a drawback for the rest of the cases.

The metrics for the ASV-like calling strategy showed an accuracy value of 96.2% and AUC of 0.964 (Fig. 2), indicating an outstanding performance in the identification of the lineages for cases with one or two SARS-CoV-2 variants. Based on these results, the ASV-like calling is a suitable strategy to identify co-infections with two SARS-CoV-2 VOCs.

Finally, we investigated the possible occurrence of SARS-CoV co-infections in Costa using the ASV-like approach. We found that none of the 1021 samples were identified with concomitant infections with distinct VOCs in the period October 12th–December 21th 2021.

## Discussion

The SARS-CoV-2 genome has rapidly evolved into multiple variants due to not only the widespread in diverse human populations but also the increase in the mutation rate during 2021^3^. In this context, the interaction of multiple viral sequences with each other during simultaneous infection can lead to potential differences in epidemiological behavior^11^. Thus, it is vital to reveal the frequency of co-infection events, how often it occurs in the population as well as and the exact composition of lineages^13^.

Here we presented an analysis of co-infection by divergent VOCs of the SARS-CoV-2 virus, in which samples with two distinct genotypes were analyzed using a metagenomic approach by ASV inference. Similar to another work by^13^, we assumed that the existence of specific lineage-defined feature mutations of the lineages in viral quasi-species achieves the identification of co-infection events (Fig. 3). A custom SARS-CoV-2 database led to identifying specific ASV belonging to VOCs, as well as non-specific ASV found in other genotypes. The metrics of the classification (VOCs classes) revealed a high performance of the pipeline with an accuracy of 96.2% and AUC of 0.964. This completely contrasted with the metagenome assembly approach, in which the classification was suggested to be by chance (accuracy = 46.2% and AUC = 0.500). The poor performance of the metagenome assembly relies on the generation of a single consensus sequence even for cases with two distinct genomes, in which a mutation of a genotype can be overshadowed by the presence of the non-mutated nucleotide in the other sequence, creating an incorrect profile of few mutations with an erroneous VOC class assignment (Table 2).

Besides, previous to the arrival of the omicron variant by November 2021, the first version of this work was prepared and the accuracy was reported at 96.3% for the ASV-like approach and 57.7% for the whole-genome assembly (more data not shown). This update demonstrated the versatility of our approach to incorporate new genotypes to infer co-infections with distinct VOCs.

Although the pipeline can be adapted to identify co-infection with more than 2 genotypes, the relevance of implementing the analysis with three 3 genotypes is questionable due to the very low incidence of co-infection with 2 genotypes (almost impossible with 3) and the unnecessary negative impact on the performance (mutations among three lineages have more chance to create a mutation profile close to another single lineage). Thus, we only considered combinations with two divergent genotypes.

Also, the ASV approach was originally designed to identify distinct bacteria using 16S rRNA. By adapting the database, this method is completely suitable to identify co-infections with other pathogens. However, for distinct microorganisms, metagenome assembly is a better strategy to identify dual infection. In our case, the SARS-CoV-2 genotypes are not different enough to use the metagenome assembly to identify concomitant infections.

Regarding sequencing data, the high reliability of Illumina technology has been reported to keep the genomic evidence of co-infections or within-host variations^13^, which has motivated its use for co-infection studies, including this work. General approaches to identify the concomitant presence of organisms can be done using: (i) metagenomics strategies, or (ii) strategies based on the reconstruction of haplotypes by mapping. For SARS-CoV-2 co-infections, the last has been the selected method due to the availability of ready-to-use bioinformatic tools^26–28^. Despite this, viral haplotype reconstruction programs usually perform poorly for sequences with low divergence or rare haplotypes^29^, which represent a possible limitation for the use among samples with simultaneous SARS-CoV-2 genomes. Because of this, the assembly of single genomes and the subsequent combination into simulated co-infection data were preferred here not only for the developed pipeline but also to create the ground-truth dataset rather than the comparison to a haplotype caller.

Using analysis of haplotype reconstruction, some studies have reported events of co-infection caused by the occurrence of two distinct genotypes. In 2020, 19 cases of co-infections were identified in Iraq^11^. Up to 8% of co-infections were informed in a study from Singapore^2^, while at least 5% was estimated in the United Arab Emirates^12^. In Brazil, a co-infection was detected with local lineages in early 2021^10^. By September 2021, a study analyzed 30,806 raw sequence datasets, in which about 2.6% were identified as co-infections with high confidence^3^.

To our knowledge, only one study has implemented a specific pipeline to identify co-infections by distinct SARS-CoV-2 genotypes, which used a strategy with an intra-host variant calling analysis and a hypergeometric distribution method^13^. Using sequencing data of COVID-19 cases from the United States of America, the authors recognized only 53 out of 29,993 samples (0.18%) as co-infection cases. A single case was reported with three lineages, while the other 52 were identified with two genotypes. These results regarding the frequency are in line with our results on the possible occurrence of co-infections in Costa Rica. None of the 1021 analyzed cases were identified as a concomitant presence of VOCs. Due to the frequency of co-infections, which is suggested to be very low, the analyzed cases could be not enough to identify at least a single case in this country. In addition, other factors affecting this result are the very few sequenced samples in comparison to the diagnosed cases (0.4% in Costa Rica and 0.41% in Latin America according to GISAID database), the inability to clinically differentiate cases of co-infection (see later), as well as the rapid displacement of circulating lineages by VOCs which have higher transmissibility. However, with the co-dominance of Delta and Omicron during the transition between the years 2021 and 2022^30^, the reports of co-infections could be increased in the coming months. Thereby, this work could be a useful tool to investigate the occurrence of this phenomenon.

Regarding the biological meaning, the report of co-infections is of concern because other studies have demonstrated that this phenomenon can contribute to the recombination of RNA viruses^1,8,13^. Product of the recombination processes, the new virions may acquire different pathogenic properties^1^ and it might impact the clinical presentation of the disease into more severe symptoms^13^. In detail, co-infections can impact viral evolution by inducing recombination and possibly generating new genotypes. In this scenario, new features regarding transmission, vaccine effectiveness, or clinical outcome can be also triggered. Thus, the contribution of this study is mainly to support genomic surveillance and eventually provide an epidemiological context to explain the possible origin of recombinants. This is an eventual first step to making a decision regarding additional boosters or developing new vaccines based on the genome architecture, as it has been previously reported for mutation-based changes.

Regarding the clinical outcome, cases with a co-infection with distinct SARS-CoV-2 genotypes have been reported with the same symptoms as other COVID-19 patients^2,10^. However, a single report of co-infection, in a young female patient without co-morbidities that presented a severe COVID-19, suggested the concomitant infection as responsible for the clinical presentation^9^. More studies and updated statistics are required to establish the relevance of co-infections in terms of severity and mortality of COVID-19 disease^11^. Also, the detection of possible cases of co-infections is another factor to consider in the interaction between the immune system and SARS-CoV-2 mutations. It has been suggested that immunity driven by a specific SARS-CoV-2 genotype does not protect against another one but can instead lead to a more severe disease pattern^9^. However, the real immunological implications of co-infections on the cellular or humoral levels are not well known^10^.

On the other hand, in this study some considerations and limitations are needed to take into account. First, the approach using ASV inference requires a custom database that needs to be built using sequences of genomes circulating locally, i.e., local genomic surveillance is a previous step to implement the pipeline. This includes the update of VOC sequences carrying new mutations (sublineages). Second, similar to the other approaches to identify co-infections, only co-infections with divergent sequences (VOCs in this case) can be identified. A test using co-infections with VOIs and VUMs was not able to identify concomitant sequences due to the low diversity, in which a poor performance was obtained during the genotype classification. Also, de novo intra-host mutation cannot be identified using this approach. This does not represent a drawback for this implementation because there is a very low probability of the de novo appearance of mutations corresponding exactly to all the feature mutations of the VOCs. If some de novo mutation was equal to a feature mutation, the ASV can be discarded with the implementation of the threshold of the mapping ASVs, as we followed here. Finally, although co-infection with SARS-CoV-2 and other pathogens have been reported, such as Influenza or bacterial agents^31,32^, and this pipeline could be adapted to identify them, a metagenome assembly could be a suitable strategy rather this approach.

Altogether, this analysis represents a new effort to track the SARS-CoV-2 genotypes circulating in Costa Rica, which are complementary to our other local studies for genomic surveillance^7,33^ as well as the identification of clinical patterns of COVID-19 patients^34^. Concomitant infection with distinct viral genotypes can lead to the generation of SARS-CoV-2 variants with possible new properties in terms of transmission, severity, mortality, or vaccine effectiveness. This remarks the relevance to continue with the surveillance of the dynamics of the pandemic including origin and tracking, genotyping, and clinical features of the infections worldwide, which can eventually arise new insights about co-infection events.

## Conclusions

In conclusion, we developed a metagenomic pipeline based on ASV-like inference for the identification of co-infection with distinct SARS-CoV-2 VOCs, in which a 96.2% of accuracy was achieved. This performance was outstanding in comparison to the whole-genome assembly approach in which a resolution by chance was suggested with an accuracy of 46.2%. The custom SARS-CoV-2 database used for the ASV-like inference can be updated according to the appearance of new VOCs to track co-infections with eventual new genotypes. In addition, the application of the ASV-like approach to all the 1021 sequenced samples from Costa Rica in the period October 12th–December 21th 2021 found that none corresponded to co-infections with VOCs. Although a small percentage of COVID-19 cases worldwide are reported as co-infections with different SARS-CoV-2 lineages, the spread of more transmissible variants and the possibility of recombination to induce new genotypes remark the need for developing tools and pipelines to track concomitant infections with SARS-CoV-2 variants. Thus, this work represents another piece in the process of the genomic surveillance of the COVID-19 pandemic in Costa Rica and worldwide.

## Supplementary Information

Supplementary Table S1.

## Data Availability

Script and the custom database used in this work are available at https://github.com/josemolina6/sars-cov-2-co-infections.
